# Association Between Plant Food Consumption and Depressive Symptoms in Adolescents: A Systematic Review

**DOI:** 10.3390/children12121617

**Published:** 2025-11-27

**Authors:** Amalia Pitsika, Ioanna Kontele, Theodoros N. Sergentanis, Stavroula-Angeliki Pantou, Eleni Kornarou, Tonia Vassilakou

**Affiliations:** 1 Department of Public Health Policy, School of Public Health, University of West Attica, 196 Alexandras Avenue, 11521 Athens, Greece; mdy20032@uniwa.gr (A.P.); ikontele@uniwa.gr (I.K.);; 2 Department of Medicine, School of Health Sciences, National and Kapodistrian University of Athens, 11521 Athens, Greece

**Keywords:** depression, adolescents, nutrition, diet, fruits, vegetables, legumes, whole grains, dietary fiber

## Abstract

**Highlights:**

**What are the main findings?**

- Consumption of plant foods is negatively associated with symptoms of depression in adolescents.
- Greater consumption of phytochemical-rich foods was associated with nearly 50% lower odds of depression.

**What are the implications of the main findings?**

- The study underscores the importance of promoting fruit- and vegetable-rich diets not only for physical health, but also as a potentially modifiable factor in supporting psychological well-being during adolescence.
- Increased consumption of plant foods could be a modifiable factor for the prevention or improvement of symptoms of depression in adolescents.

**Abstract:**

Background: Diet is a crucial factor in both physical and mental health. Several studies in adult populations report that a healthy diet is negatively associated with depression; however, data among adolescents are scarce. Aim: The investigation of the correlation between the consumption of plant foods and depressive symptoms among adolescents. Materials and Methods: A systematic review of articles published over the last decade was conducted across five databases: PubMed, Cochrane, Embase, Scopus, and Google Scholar. Results: Fourteen studies were included in the analysis. Twelve studies reported a negative correlation between symptoms of depression in adolescents and consumption of certain plant foods. Two studies did not report significant correlations, while one study reported a weak positive correlation. Conclusions: Studies report that the consumption of plant foods and specific nutrients included in them is negatively associated with symptoms of depression in adolescents. Further research is needed to explore the relationship and the possible biological mechanisms between plant food consumption and depressive symptoms.

## 1. Introduction

The World Health Organization (WHO) states that mental health is essential to overall health and well-being. Disability, morbidity, and mortality rates are disproportionately higher in individuals with mental disorders [1,2]. Adolescence is a critical developmental period for mental health, with approximately half of all mental disorders emerging before late adolescence. According to UNICEF, in 2019, the prevalence of mental disorders in children and adolescents aged 10–19 was 16.3% in Europe and 13.2% globally. In Europe, suicide is the second leading cause of death among adolescents aged 15–19, with depression and anxiety accounting for 55% of mental disorders [3]. WHO predicts that depression will be the leading cause of illness and disability worldwide by 2030 [4]. Depression is a significant factor in suicide, which is the fourth leading cause of death among adolescents aged 15–19 years globally [5].

Depression among adolescents may have serious consequences requiring long-term treatment, making the mental health of children and adolescents a public health priority. Failing to address the mental health problems of children and adolescents promptly will inevitably have serious consequences on the adult population of the future. Recognizing the healthcare and economic burden of mental illnesses, public health initiatives are focusing on prevention by identifying modifiable factors that promote mental health and prevent depression.

Lifestyle modifications in diet and physical activity have increasingly been reported to not only prevent, but also reduce the symptoms of depression. Both diet and physical activity are modifiable and cost-effective factors in preventing depression, as well as supplementing modifiable options to current treatments [6,7,8].

The quality of diet has been reported to be correlated to the risk of depression. While depression is often associated with biochemical or emotional dysfunctions, diet can play a decisive role in the initial onset, as well as the severity and duration of depression [6,9,10,11]. Nutritional Psychiatry is a new field of research, which shows great promise in the prevention and treatment of depression as well as other mental disorders. The studies in the field focus on understanding the biological mechanisms underlying the relationship between diet and depression, exploring how dietary interventions may have a direct impact on various biological systems and mechanisms associated with depression, such as the immune system, oxidative stress, brain plasticity, chronic inflammation, and the brain–gut microbiome axis [12,13].

Recent systematic reviews and meta-analyses, mainly in adults, suggest an inverse correlation between a healthy, especially plant-based, diet and depression. A diet rich in fruits, vegetables, fish, and whole grains may reduce the risk of depression in adults. Studies have shown that every 100 g increase in daily fruit and vegetable consumption correlates with a 3% reduced risk of depression in prospective studies, and a 5% reduction in cross-sectional studies [14,15,16,17]. The Mediterranean Diet, which is based on a high daily consumption of fruits, vegetables, whole grains, legumes, nuts, fish, white meat, and olive oil, has also been negatively correlated with depression [18].

Positive correlations between healthy diets and mental well-being of children and adolescents have also been reported [17,19,20].

Nevertheless, further research is required to examine the relationship between diet and depression in adolescents, focusing on specific types of foods, combinations of foods and the amount that teenagers should consume in order to maintain or improve their mental health. To date, systematic reviews and meta-analyses have linked healthy eating patterns and a diet rich in fruits and vegetables to reduced depression risk, but these studies have primarily focused on adults. There are not enough systematic reviews or meta-analyses on adolescent populations, and there has been no research on the broader range of specific plant foods (fruits, vegetables, whole grains, legumes, vegetable oils, and nuts), which significantly contribute to healthy nutrition in relation to depression in adolescents. Therefore, the present systematic review aims to investigate the association between the consumption of plant foods and depressive symptoms in adolescents.

## 2. Materials and Methods

### 2.1. Literature Search Strategy

A systematic review of articles published in English over the last decade was conducted across five electronic databases: PubMed, Cochrane, Embase, Scopus, and Google Scholar. This search was performed according to the 2020 PRISMA guidelines [21]. The systematic review has been registered at OSF (registration number Q76ru). The terms listed in Table 1 were combined to find studies relevant to the review topic. Additionally, Table 2 displays the PICOS algorithm that was used.

### 2.2. Eligibility Criteria

As indicated in Table 3, articles that met the inclusion criteria were eligible for inclusion in this review. Two authors (P.A., and S.-A.P.) blindly screened all article abstracts. Those who did not comply with the inclusion criteria were eliminated, and any disputes were resolved by consensus in a meeting, where the abstracts were reviewed.

### 2.3. Quality Assessment

The Newcastle–Ottawa scale (NOS) and its versions, adapted to assess the quality of non-randomized cross-sectional, case–control, and cohort studies, were used to rank each study. This scale gives comparability, outcome (assessment, statistical test) and selection (representativeness, sample size, nonrespondents, and ascertainment of exposure) a maximum of 10 stars. The updated Cochrane ROB2 tool, which evaluates five factors—random sampling, intervention methodology, missing data, outcome assessment, and presentation of results—was used for interventional studies. To minimize potential bias and address the need for a rigorous methodology, two reviewers conducted the quality assessment for all included studies independently. All disagreements in the quality assessment judgments were systematically resolved through a documented consensus process to finalize the assessment for all included studies. Discrepancies between the two reviewers were first discussed and resolved through verbal and documented consensus. For any remaining disagreements that could not be resolved between the initial two reviewers, a third, independent reviewer was consulted. The third reviewer’s judgment was considered decisive, resulting in the final agreed-upon risk of bias rating for that study or domain. The final detailed quality assessment of the studies included is presented in the Complementary Material.

### 2.4. Data Collection Process

Data were extracted from each study using a structured coding scheme in Microsoft Excel. They included the name of first author, the year of publication, the country, the study design, the sample size, and the age of adolescents. Moreover, the instruments used to assess eating habits, and to evaluate depressive symptoms were recorded. Finally, the association between exposure (eating behavior) and impact (depressive symptoms) was assessed.

### 2.5. Compliance with Ethics Guidelines

This article is based on previously conducted studies. The study is performed in accordance with the Preferred Reporting Items for Systematic Reviews and Meta-Analysis (PRISMA) guidelines [21].

## 3. Results

### 3.1. Eligible Studies

**Figure 1:**
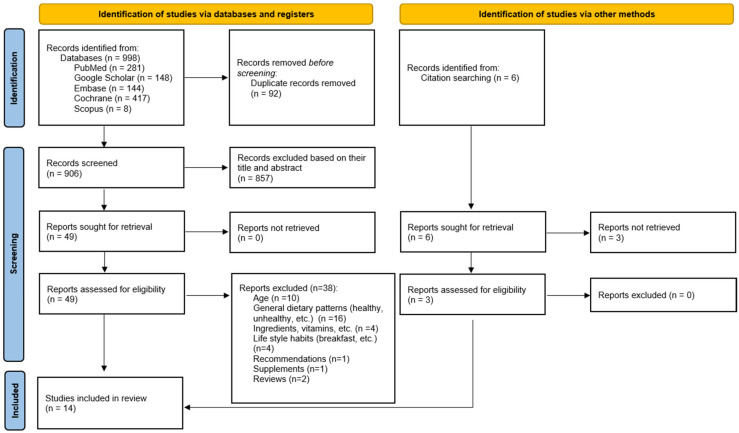
Flow diagram of the study selection process.

Data obtained from online databases yielded 998 studies and 6 from other sources. Following the removal of 92 duplicates, we examined the remaining papers and discarded 857 because their titles and abstracts did not align with the study’s objective. Following the retrieval of 52 full articles, 38 articles were eliminated based on the inclusion and exclusion criteria. Ultimately, 14 studies that met the eligibility criteria were included in this systematic review. A PRISMA 2020 flow chart describing the sequential steps is presented in Figure 1.

### 3.2. Characteristics of Eligible Studies and Population

Of the 14 studies included in the systematic review, ten were cross-sectional, one case–control and two prospective studies, while one was an intervention study. Three studies were conducted in Europe (two in the United Kingdom and one in Norway), nine in Asia (four in North Korea, two in China, two in Iran and one in Japan), and two in Australia. The sample sizes of the studies ranged between 64 (intervention study) and 244,250 individuals. The mean age of the participants ranged from 12.7 to 17.5 years old. Characteristics and results of the eligible studies are presented in Table 4.

### 3.3. Prospective Studies

Two prospective studies were included in this systematic review. Winpenny et al. (2018) [22] found no significant correlations between diet quality, fruit and vegetable consumption, and symptoms of depression, neither at baseline nor during the three-year follow-up. Only at age 14, a negative link between fruit and vegetable consumption and depression score was observed in the unadjusted model. Still, adjustment for behavioral factors, including smoking and alcohol consumption, reduced this correlation [22]. Contrary to these results, Swann et al. (2021) [23] reported that adolescents with the highest intake of total dietary fiber from fruits, vegetables, and whole grains have significantly lower odds of presenting moderate/extreme symptoms of depression compared to adolescents with the lowest intake of fiber. However, it should be noted that the effect of dietary fiber does not occur independently of other nutrients [23].

### 3.4. Cross-Sectional Studies

Ten cross-sectional studies were included in the systematic review. Nine reported a negative correlation between the consumption of plant foods such as fruits, vegetables (mainly green and yellow), whole grains (bread, cereals, refined rice), legumes (beans), and/or plant food components such as fiber, vitamins, trace elements, and antioxidants contained in plant foods and symptoms of depression in adolescents [24,25,26,27,28,29,30,31,32].

A study by Yim et al. (2021) in North Korea found that mental health issues (depression, stress, suicide attempts) were negatively correlated to fruit and vegetable intake [24]. Previously, Hong and Pletzer (2017) [25] also reported 14% and 22% lower risk of depression for those eating fruits and vegetables twice a day. At the same time, unhealthy eating behaviors were positively correlated with symptoms of depression. However, it remains unclear whether unhealthy eating habits are the underlying cause or a consequence of depression [25].

In a study of Japanese students, Tanaka and Hashimoto (2019) [26] found a strong inverse relationship between regular green and yellow vegetable intake and depressive symptoms in both middle school and high school students. Moreover, adolescents who consumed green and yellow vegetables one or more times per day had significantly fewer depressive symptoms than those who either never consumed vegetables or consumed them only 1–2 times per week [26]. Accordingly, Lv et al. (2022) found that adolescents who ate fresh fruits more than once a day and vegetables two or more times a day reported fewer symptoms of depression than adolescents who did not eat fruits and vegetables on a daily basis [27]. Similar findings were reported by Smout et al. (2023) [28], who found a lower likelihood of symptoms associated with depression in Australian adolescents who had higher fruit and vegetable consumption. Specifically, the lowest depression symptom score was observed among adolescents who had three servings of fruits per day (37% lower than those who had less than one serving per day). In addition, participants who had two servings of vegetables had 34% lower depression symptom scores than those who consumed less than one serving per day [28].

In Norway, Kleppang et al. (2021) [29] reported a negative correlation between the consumption of healthy foods, such as fruit and whole grain bread, and the prevalence of any symptoms of depression among students. Furthermore, a positive correlation was reported between the consumption of unhealthy foods and drinks and the prevalence of any symptoms of depression. However, the results also reported a weak positive correlation between vegetable consumption and symptoms of depression [29]. The most plausible explanation for this weak association is reverse causality. More specifically, individuals who are experiencing sub-clinical depressive symptoms or who have recently received a depression diagnosis may be actively attempting to improve their mood and health through conscious dietary changes, such as increasing their vegetable intake. In this case, the elevated vegetable consumption is the result of the depressive state, not the cause. Another potential explanation may be the association with other possible confounders, such as social competence, family cohesion, and appearance satisfaction, which were not included in the study [29].

Liang et al. (2022) [30] found that fruit consumption was linked to a lower risk of depression, but only in girls. No significant association between vegetable consumption and depression was reported after adjustment for confounders. The varying roles of fruit and vegetable consumption observed in the study also provide a rationale for examining these two food groups separately in the future [30].

Khayyatzadeh et al. (2021) [31], in a study of adolescent girls in Iran, revealed a negative association between dietary intake of specific antioxidant carotenoids (β-carotene, α-carotene, and lutein) and depression among adolescent girls. In addition, higher dietary intakes of both soluble and insoluble fiber had a positive effect on both the symptoms and severity of depression. However, due to the nature of the study, it was not determined whether the development of depression leads to lower consumption of antioxidant-rich foods or whether low dietary intake of antioxidants and fiber contributes to the development of depression [31]. Similarly, according to Sangouni et al. (2022) [32], the prevalence of depression is significantly reduced when the DPI phytochemical index score increases. A higher phytochemical index (DPI) score negatively correlates with the risk of depression. Eating foods such as fruits, vegetables, legumes, nuts, and whole grains significantly increases phytochemical index (DPI) score. Adolescent girls who consumed high DPI foods were 50% less likely to be depressed compared to those who consumed low phytochemical index (DPI) foods. This relationship remained significant even after adjustments for age, energy intake, BMI, physical activity, parental marital status, and parental death were made [32].

Finally, contrary to the previous studies, the study of Park et al. (2018) on adolescents in South Korea reported that healthy eating behaviors such as increased fruit and vegetable consumption were not linked to depression, but only to higher odds of good general and oral health, emotional state, and adequate sleep in adolescents [33].

#### 3.4.1. Case–Control Study

In the study by Kim et al. (2015) [34], the risk of depression had a significant negative association with consumption of at least three servings of green vegetables per day, one to three servings of fruit per day, unprocessed rice, and beans. Green vegetable consumption was more prevalent in the group with no signs of depression. In addition, the risk of depression was negatively correlated to consumption of fiber, most micronutrients such as beta-carotene, vitamin B (B1, B2, B6, B12), vitamin E, vitamin C, potassium, zinc, folate, iron, magnesium, and copper. In contrast, the risk of depression was reported to be associated with fast food and processed food consumption [34].

#### 3.4.2. Intervention Study

The results of the intervention study of Fisk et al. (2020) [35] in the United Kingdom showed that daily consumption of wild blueberry in the form of drink is beneficial in reducing symptoms of depression in adolescents. The study results suggest that flavonoid interventions may have the potential to reduce depression in adolescents [35].

#### 3.4.3. Quality Assessment of the Reviewed Studies

Based on the study design, the quality of the examined studies was evaluated using the appropriate instruments. Cross-sectional, case–control, and cohort studies received good or high quality ratings. The one interventional study received a low risk of bias rating (Supplementary Tables S1–S3).

## 4. Discussion

The aim of the current systematic review was to identify and analyze various studies investigating the association between plant food consumption and depressive symptoms in adolescents. Fourteen studies met the inclusion criteria. The qualitative evaluation of the studies revealed that the studies were of high quality.

Twelve studies [23,24,25,26,27,28,29,30,31,32,34,35] reported inverse association between the consumption of certain plant foods—such as fruits, vegetables, whole grains and beans—and depressive symptoms in adolescents. On the contrary, two studies [22,33] did not observe significant correlations.

Several studies [22,24,25,26,27,28,29,30,33,34] examined the frequency or quantity of fruit and vegetable intake, with some suggesting that adolescents consuming at least two servings of fruits and two to three servings of vegetables daily had lower odds of reporting depressive symptoms. Findings from previous large-scale reviews also support these findings. Liu et al. (2020) found that consuming fewer than five servings of fruits and vegetables per day was associated with a higher likelihood of depressive symptoms among adolescents from low and middle income countries [36]. Accordingly, a systematic review of cohort studies in young people supported the evidence that fruit consumption may be associated with decreased risk of developing depression [17]. Moreover, a systematic review revealed that higher dietary fiber intake was associated with approximately 49% lower odds of depression in children and adolescents compared to lower intakes [37].

Beyond general fruit and vegetable consumption, several studies [31,32,35] emphasize the potential role of specific micronutrients and bioactive compounds. For example, higher dietary intake of carotenoids and lutein was inversely associated with depressive symptoms [31], while greater consumption of phytochemical-rich foods was linked to nearly 50% lower odds of depression [32]. Furthermore, a small intervention study suggested that daily consumption of wild blueberry drinks was associated with reduced depressive symptoms in adolescents [35]. These findings imply that diet–depression pathways may be mediated in part by the antioxidant and anti-inflammatory properties of plant-based compounds. Nutrients and bioactive compounds are associated with improved mental health through the following mechanisms: counteracting oxidative stress and inflammation, modulating neurotransmitters, acting against mitochondrial dysfunction, supporting neuroplasticity, and influencing the brain–gut axis [38]. More specifically, bioactive compounds (phytochemicals) have significant health effects, often acting as potent antioxidants and anti-inflammatory agents regulating redox balance, which is a key factor in modulating depressive symptoms. Chronic low-grade inflammation and oxidative stress are strongly implicated in the pathology of depression [39]. Compounds like polyphenols and minerals like zinc support the creation of new neural connections and the overall health and function of brain cells, often by increasing brain-derived neurotrophic factor (BDNF) [40,41]. In addition, phytochemical substances are reported to enhance mitochondrial biogenesis and modulate apoptosis, while also reducing neuroinflammatory cytokines and normalizing HPA axis activity [42]. Furthermore, B vitamins, iron, and zinc are direct or indirect co-factors in the synthesis and regulation of mood-critical neurotransmitters (serotonin, dopamine, norepinephrine) [43]. Moreover, dietary fibers and specific polyphenols modulate the composition and function of the gut microbiome which, in turn, influences the production of neuroactive compounds and regulates the immune and nervous systems linked to mood [44].

Contrary to the results of the current review, Dabravolskaj et al. (2022) reported that prospective studies have not consistently identified significant correlations between fruit and vegetable consumption and depression in adolescents [45]. Similarly, Jain et al. (2020) found mixed evidence regarding plant-based diets and symptoms of depression [46].

In the current review, most of the data concerned the consumption of fruits and vegetables. Only a few studies [23,29,31,32,35] addressed other plant foods, such as legumes, beans, whole grains, unprocessed rice, grains, or specific types of fruit (wild blueberry), specific types of vegetables (green and yellow), or their ingredients, such as fiber and antioxidants or a phytochemical indicator. No studies were found on the consumption of seeds, olive oil and other vegetable oils. One study documented the correlation between depression and overall mental health and diet (negative inverse correlation), questioning whether less healthy eating behavior is the cause or consequence of depression. Therefore, further investigation of the correlation between a plant-based diet and depression in adolescents is warranted.

In addition, it is worth mentioning that across studies, a higher prevalence of depressive symptoms was generally observed among teenagers who reported greater consumption of fast food and processed foods, were older, were in a significantly poorer financial situation, and were girls. In addition, other lifestyle behaviors could also be associated with the risk of developing symptoms of depression and may be considered as confounders. Adolescents who reported healthier lifestyle behaviors, such as daily consumption of breakfast, enough sleep, avoidance of alcoholic beverage and sugary drinks, frequent participation in moderate to vigorous physical activity, limited daily screen time, and avoidance of smoking tended to exhibit lower depression scores.

Furthermore, differences associated with sociodemographic characteristics have been identified. More specifically, the majority of studies indicated that a significantly higher proportion of girls reported a high level of depressive symptoms compared with boys [22,23,26,27,28,29,30]. Additionally, a greater prevalence of depressive symptoms among girls than boys was observed at ages 14 and 17 in both prospective studies [22,23]. Girls with irregular menses had more depressive symptoms compared to those with regular menses [34].

Adolescents tend to develop depressive symptoms at different ages. The depressive symptoms’ score increases with the adolescents’ age. High school students show significantly higher levels of depressive symptoms than junior high school students [23,26,29]. In contrast to a significant body of research indicating that the prevalence of these conditions increases during adolescence, Smout et al. (2023) reported a weak negative association of older age with depression [28].

There is also a gender-related association in dietary intake. Females more frequently consume breakfast and fruits, whereas males more frequently consume vegetables, fast food, and carbonated and caffeinated beverages [24]. Eating fruits and breakfast is associated with significantly lower risks of mental health problems. The association between eating fruit and mental health is stronger in females [30]. These results provide fundamental data that can be used to develop and implement effective nutrition intervention programs for adolescents.

Moreover, the studies indicate differences between adolescents with lower socioeconomic status (SES) and those who have higher SES. Adolescents from families with higher SES have lower odds of experiencing depressive symptoms. In contrast, adolescents with a high level of depressive symptoms tend to have a significantly poorer family income [23,28,29]. Family is an important factor for adolescents’ mental health. Those with the highest relative family affluence report significantly lower depression scores compared to those with the lowest [28]. Moreover, adolescents with moderate to severe depressive symptoms often have parents with no formal education and a history of mental health problems [23].

Moreover, an association between cultural and linguistic diversity (CALD) and mental health has been revealed. CALD adolescents have significantly higher levels of mental health problems than non-CALD adolescents. Cultural and linguistically diverse (CALD) females show significantly higher depressive symptoms than males [28].

Furthermore, the studies support previous findings that adolescents in low socioeconomic positions are more likely to have poor diet quality [30]. The poorer the economic situation, the poorer the dietary intake. Specifically, groups perceiving their economic status as low report lower consumption of breakfast, vegetable, and milk but higher consumption of fast food and carbonated and caffeine beverages. The higher the economic status, the higher the breakfast, vegetable, milk, and fruit intake [24]. These findings support previous evidence that adolescents who live in economically disadvantaged areas and have a low socioeconomic status are more likely to have poor diet quality [30]. There were no statistically significant differences in depression scores between adolescents in regional areas and adolescents in major city areas (Smout, 2023 [28]).

Students attending schools with the highest index of socio-educational advantage have significantly lower depression scores [28]. Additionally, students with high academic performance tend to more frequently consume breakfast, vegetables, milk, and fruit while consuming fewer carbohydrate beverages. On the other hand, students with lower academic achievement more frequently consume fast food and carbohydrate and caffeinated beverages [24].

There are minor gender differences in the associations between various lifestyle factors and depressive symptoms [29]. Adolescents with moderate or severe depressive symptoms appeared to have poorer lifestyle habits, with lower physical activity and higher rates of binge drinking [23]. Overall, adolescents who are more physically active and obtain sufficient sleep consume breakfast, vegetables, fruits, and milk more regularly [24].

Regarding the association between dietary intake and Body Mass Index (BMI), adolescents who have obesity tend to consume carbohydrates and caffeinated beverages more frequently while consuming fewer vegetables and less breakfast [24]. Numerous studies have confirmed the association between dietary intake and obesity.

While most studies used adjusted statistical models for age, socioeconomic status, energy intake, BMI, alcohol consumption, smoking, physical activity, and sleep, and some of them also controlled for more potential confounding variables such as adiposity, residency, family structure and functioning, and parental death or divorce, the impact of the main confounding factors on the association between plant food consumption and depressive symptoms was not always significant.

The observed association between plant food consumption and reduced depressive symptoms highlights a critical opportunity for proactive mental health promotion through public health policy, particularly within the school environment. Schools are ideal settings for interventions, as they reach children and adolescents during a crucial period of brain development and habit formation, and before the full onset of many chronic mental health issues [47]. Efforts should be directed toward actions that address both the short- and long-term significance of attaining a healthy profile, free of metabolic risks and psychological disorders [48]. Integrating dietary interventions focused on plant-rich foods can serve as a primary prevention strategy, complementing traditional mental health services.

Relevant strategies for school-based interventions may include nutrition education and curriculum integration, implementation of actions aiming at school cafeterias and food environment policies which would prioritize the availability and accessibility of diverse, nutrient-dense plant foods, and parental and community engagement through workshops and resource distribution. Successful integration of these dietary strategies is expected to yield dual benefits for public health: reduced mental health burden and long-term health dividends [48]. This approach would position dietary quality not merely as a physical health concern, but as a fundamental pillar of a holistic, prevention-focused public mental health strategy.

The present systematic review has several limitations that should be mentioned. First, the number of included studies was small. Only 14 studies have been found to investigate the correlation between plant food consumption and depression in adolescents, making it difficult to draw conclusions. Also, most of the included studies were cross-sectional, which does not allow for the formulation of conclusions regarding possible causal relationships between the studied variables. Regarding prospective studies, it is also unlikely to determine causal relationships. This does not permit addressing and clarifying the issue of potential bidirectionality—whether poor diet causes depression or vice versa. Symptoms of depression can be considered an effect, but also an exposure factor affecting lifestyle and, thus, also diet. In addition, most food frequency and mood data have been collected through self-report questionnaires rather than interviews, which are prone to recall bias and subjective misclassification. Moreover, heterogeneity in dietary assessment tools, depression scales, and confounding variable control (e.g., socioeconomic status, physical activity, and sleep) restricts cross-study comparability. Notably, the high-versus-low consumption categories were defined inconsistently across the included studies; as their contrasts did not correspond to the same exposure increment, no quantitative synthesis (meta-analysis) was undertaken. The limited geographic diversity—heavily weighted toward Asian populations—also reduces generalizability. Moreover, the absence of studies on specific plant food categories (e.g., nuts, seeds, vegetable oils) and the lack of biomarker-based validation (e.g., serum carotenoids or polyphenols) further constrain mechanistic interpretation. Additionally, it is worth mentioning that it is difficult to isolate the association of a single food or nutrient with symptoms of depression, making it fallacious to rule out a confounding effect of other foods and/or nutrients present in the diet. Lastly, the exclusion of studies with mixed dietary patterns may have introduced bias, whereas the inclusion of only English-language publications may have resulted in language bias and decreased global representativeness. For the above reasons, the results should be interpreted with caution.

## 5. Conclusions

In conclusion, the available evidence indicates that the consumption of plant foods, such as fruits, wild blueberries, vegetables, yellow and green vegetables, whole grains, unprocessed rice, legumes, beans, and, in general, the fiber and antioxidants contained in these foods, is generally associated with fewer symptoms of depression in adolescents. Also, higher dietary photochemical index scores appear to be linked with lower levels of depressive symptoms.

The convergence of evidence suggests that fruit- and vegetable-rich diets may play a supportive role in adolescents’ mental health, in addition to their established benefits for physical health. Emphasizing the consumption of plant foods could represent a potentially modifiable factor associated with fewer symptoms of depression in adolescents.

Furthermore, exploring the potential moderating roles of gender, socioeconomic status, physical activity, energy intake, sleep, and psychosocial factors through validated measures in the future will also be essential to advancing a more nuanced understanding of how diet relates to adolescent mental health.

While current evidence does not permit causal interference, such dietary patterns may be considered within broader public health policy and initiatives aimed to promote healthy behaviors and mental well-being of adolescents, particularly by implementing relevant public health activities within the school environment.

## Figures and Tables

**Table 1:** Keywords for the PubMed database.

**Search string** (fruit* OR vegetable* OR ‘whole grain*’ OR ‘dietary fiber*’ OR ‘plant foods’ OR legumes OR diet OR nutrition OR seeds OR antioxidants OR ‘olive oil’ OR ‘plant oils’ OR ‘seed oils’ OR nuts) AND depression AND adolescents.

**Table 2:** PICOS algorithm for systematic review.

Participants	Intervention	Comparison	Outcomes	Study Design
Adolescents 10–19 years old	Consumption of plant foods (fruits, vegetables, whole grains, legumes, olive oil, vegetable oils, nuts and seeds)	High consumption of plant foods compared to low or no consumption of plant foods	Depression/symptoms of depression	Primary studies (cross-sectional studies, prospective cohort studies, intervention studies, patient-control studies)

**Table 3:** Inclusion and exclusion criteria.

**Inclusion Criteria**
1. Participants are adolescents between 10 and 19 years old (healthy or with symptoms of depression or diagnosed with depression). 2. Participants are eating plant foods (fruits, vegetables, whole grains, legumes, olive oil, vegetable oils, nuts and seeds) 3. Data about the correlation between plant food consumption and depression or depressive symptoms should be provided 4. Studies that included both children and adolescents, as well as adults, were included in the review only if they provided separate data for adolescents. 5. Any strategy to diagnose depression or depressive symptoms and to assess plant foods consumption was deemed eligible. Regarding the instruments used to evaluate depression, depressive symptoms, or plant food consumption, accepted studies were considered those that used validated questionnaires or were adapted from validated questionnaires. 6. Prospective cohorts/cross-sectional/case–control and interventional (clinical trial) studies were included. 7. Regarding the interventional studies, only studies that provided information on depression scores prior to and following the intervention were included. 8. The articles were written in the English language. 9. Studies were published between January 2015 and February 2025.
**Exclusion Criteria**
1. Case reports. 2. Review articles and medical hypotheses. 3. Animal studies. 4. Studies that did not report primary data (simple and systematic reviews, meta-analyses, commentaries, etc.). 5. Studies not declaring age groups. 6. Studies with younger children (less than 10 years old) or adults (more than 19 years old). 7. Studies on adolescents with other health problems or mental disorders other than depression. 8. Studies investigating dietary patterns with mixed food groups (e.g., fruits, vegetables and fish in the same food group) and/or with nutritional supplements. 9. Studies that used questionnaires that do not assess depression or depressive symptoms. 10. Studies not published in English.

**Table 4:** Characteristics and Results of the Eligible Studies.

	Study, Country	Type of Study	Sample Size	Age	Exposure Assessment	Impact Measurement	Confounders	Key Findings
1	Winpenny et al., 2018 [22], United Kingdom	Prospective	N = 603 (40.0% boys)	Mean age = 14.5 (SD = 0.3) years old and 17.5 (SD = 4.1) years old	Dietary diary for 4 days (2 weekdays and 2 weekend days)	Moods and Feelings Questionnaire (MFQ)	Adjustment for sociodemographic (age, sex, socioeconomic status SES), BMI, behavioral factors (smoking, alcohol, physical activity, sleep), psychosocial factors (friendship quality, self-esteem, family functioning), medication use, energy intake	No prospective associations were found between fruit and vegetable intake and later depression score (β = 0.14, 95% CI −0.15, 0.43). At age 14 a negative cross- sectional association between fruit and vegetable intake and depressive symptoms was found (β = −0.35, 95% CI = −0.65, −0.05) but after adjusting for behavioral covariates, the association was no longer significant
2	Swann et al., 2021 [23], Australia	Prospective	N = 1913	14 years old and 17 years old	Food Frequency Questionnaire Commonwealth Scientific and Industrial Research Organization (CSIRO)	Beck Depression Inventory for Youth (BDI-Y).	Adjustment for age, sex, energy intake, dietary misreporting, adiposity, family factors (parental education, income, history of mental health, functioning), lifestyle factors (binge drinking, leisure time, physical activity), dietary patterns (healthy and Western), inflammation.	The odds of moderate/high symptoms of depression were lower in the quartile with the highest fiber intake compared to the quartile with the lowest intake (OR 0.273; 95% CI 0.09, 0.81).
3	Yim et al., 2021 [24], North Korea	Cross-sectional	N = 187,622 (51.5% boys)	12–18 years old	Food Frequency Questionnaire	Questionnaire of Experiences with depression and suicide attempts	No confounding factors were examined.	Negative correlation between depression and consumption of vegetables (OR = 0.932, 95% CI = 0.884–0.982, *p*-value 0.001) and fruits (OR = 0.984, 95% CI = 0.948–1.021, *p*-value 0.391)
4	Hong and Pletzer, 2017 [25], North Korea	Cross-sectional	N = 65,212 (51.8% boys)	12–18 years old (Mean = 15.1, SD = 0.02)	Questionnaire assessing eating behaviors	Questionnaire for evaluation of depression, anxiety (last 12 months), health, happiness and sleep	Adjustment for sociodemographic factors (age, gender, socioeconomic status (SES), geolocality, school level), physical activity, substances’ use (alcohol, tobacco)	Negative correlation between depressive symptoms and consumption of - fruits, twice a day vs. eating no fruits (OR = 0.86, 95% CI, 0.78, 0.94) - vegetables, twice a day vs. eating no fruits (OR = 0.78, 95% CI, 0.70, 0.86)
5	Tanaka and Hashimoto, 2019 [26], Japan	Cross-sectional	N = 858 (45.3% boys)	Mean age = 15.49 years old (SD = 1.78)	Food Frequency Questionnaire (FFQ)	Depression Scale (CES-D) of Center for Epidemiological Studies	Control for age, sex, sleep duration	Significant negative correlations between regular green and yellow vegetable consumption and symptoms of depression in both middle school and high school students (r = −0.15, 95% CI: −0.24, −0.06, *p* < 0.01, r = −0.11, 95% CI: −0.21, −0.02, *p* < 0.05, respectively). Adolescents who consumed green and yellow vegetables every day had significantly fewer symptoms of depression than those who ate them never or 1–2 times a week (F (2, 853) = 4.82, *p* < 0.01).
6	Lv et al., 2022 [27], China	Cross-sectional	Ν = 6251 (47.2% boys)	11–19 years old (Μean = 14.7, SD = 1.74)	Food Frequency Questionnaire	Depression Scale of Center for Epidemiologic Research	No confounding factors were examined.	Negative correlation between fresh fruit and vegetable consumption and symptoms of depression (*p* < 0.001, χ^2^/t = 71.527, *p* < 0.001, χ^2^/t = 50.598)
7	Smout et al., 2023 [28], Australia	Cross-sectional	N = 6185 (49.3% boys)	Mean age = 12.7 years old (SD = 0.5)	Student Physical Activity and Nutrition Survey (SPANS) Questionnaire	Depression-Patient Health Questionnaire for Adolescents Scale (PHQ-A) Stress—scale PROMIS Anxiety Pediatric (PROMIS-AP)	Adjustment for age, gender, linguistic diversity (CALD) status and relative family affluence and clustering at the school level	Higher fruit and vegetable consumption correlated to less depression symptoms (F7.5548 = 13.31, *p* < 0.001 for fruits and F7.5597 = 10.72, *p* < 0.001 for vegetables). The lowest mean symptom scores were observed in participants who consumed - three servings of fruit in a typical day, average depression symptom score 37% lower (ΔMscore: 2.5, 95% CI: 1.9–3.2) than those who consumed less than one serving of fruit in a typical day - two servings of vegetables in a typical day, average depression symptom score 34% lower (ΔMscore: 2.3, 95% CI: 1.6–3.1) than those who consumed less than one serving of vegetables in a typical day
8	Kleppang et al., 2021 [29], Norway	Cross-sectional	Ν = 244,250 (47.8% boys)	13–19 years old	Questionnaire assessing Nutrition, Physical Activity, Social Media Use, PC/Tablet/Mobile Gaming, Alcohol and Tobacco Use	Hopkins Symptom Checklist 90 Questionnaire/Scale (past week)	Adjustment for age, perceived family economy, parental higher education)	Negative correlation between depressive symptoms and consumption of - fruit every day or more: OR = 0.92, 95% CI = 0.88–0.96, *p* < 0.001 (girls) - whole grain bread: OR = 0.89, 95% CI = 0.85–0.92, *p* < 0.001 (girls) OR = 0.91, 95% CI = 0.87–0.96, *p* < 0.001 (boys) Positive correlation between depressive symptoms and consumption of vegetables every day or more: OR = 1.09, 95% CI = 1.05–1.14, *p* < 0.001, (girls) OR = 1.11, 95% CI = 1.04–1.17, *p* < 0.01 (boys)
9	Liang et al., 2022 [30], China	Cross-sectional	N = 3314 (43.9% boys)	12–17 years old (Mean boys = 14.21, SD = 0.96, girls = 14.14, SD = 0.94)	Questionnaire -Subscale of the Chinese version of the Health Promoting Lifestyle Profile-II (HPLP-II)	Patient Health Questionnaire-9 (PHQ-9)—Depression Scale, Insomnia Scale, YSIS Generalized Anxiety Disorder Scale-7 (GAD-7)	Analyses were stratified by sex. Adjustment for age, grade, sibling status, parental education, family structure and income)	The frequency of eating fruits was significantly inversely correlated to the severity of depression in females: - Never eating fruits: RR = 1.34, 95% CI = 1.12–1.62, *p* = 0.002 - Sometimes eating fruits: RR = 1.18, 95% CI = 1.01–1.37, *p* = 0.033 The correlation of eating vegetables and depression severity was not significant in both sexes.
10	Khayyatzadeh et al., 2021 [31], Iran	Cross-sectional	N = 988 girls	12–18 years old (Mean = 14.5, SD = 1.54)	Food Frequency Questionnaire (FFQ) for assessment of dietary intake. Energy and nutrient intake was estimated using Nutritionist IV software	Beck Depression Inventory (last 2 weeks)	Adjusted for age, energy intake, menstruation, family members, parental death, parental divorce, BMI, physical activity	Subjects with no or minimal symptoms of depression had significantly higher dietary intakes of α-carotene (*p* = 0.01), β-carotene (*p* = 0.006), lutein (*p* = 0.03), vitamin C (*p* = 0.04) and dietary fibers (*p* < 0.001) compared to subjects with mild or severe symptoms of depression. OR (95% CI) of depressive symptoms for the highest v. lowest quartile of nutrient intakes were as follows: - 0.61 (95% CI 0.37, 1.01) for vitamin C - 0.42 (95% CI 0.26, 0.69) for β-carotene - 0.50 (95% CI 0.31, 0.79) for α-carotene - 0.71 (95% CI 0.44, 1.15) for lutein - 0.51 (95% CI 0.32, 0.82) for soluble dietary fiber - 0.42 (95% CI 0.25, 0.68) for insoluble dietary fiber
11	Sangouni et al., 2022 [32], Iran	Cross-sectional	N = 733 girls	12–18 years old Mean age = 14.5 years old	Food Frequency Questionnaire (FFQ), Dietary phytochemical index (DPI) score	Beck Depression Inventory (BDI) Quality of Life (SF-12v2)	Adjustment for age, energy intake, BMI, parental divorce or death, physical activity	Higher dietary phytochemical index (DPI) score correlated to 50% lower odds of depression compared to lower score (OR: 0.50; 95% CI: 0.30–0.84, *p* = 0.009)
12	Park et al., 2018 [33], North Korea	Cross-sectional	N = 65,529 (51.6% boys)	12–18 years old	KYRBS-2016 Questionnaire (Korean Youth Risk Behavior Survey)	Questionnaire for physical and mental health variables	Adjustment for sex, school grade, residency, socioeconomic status (SES), other dietary behaviors	Fruits >= 1 times/day had no significant effect on depressive mood (OR = 1.03, 95% CI 0.99–1.08); however, there was a positive significant association with perceived general health (OR = 1.10), perceived happiness (OR = 1.17), and perceived sleep satisfaction (OR = 1.13). Vegetables >= 3 times/day had no significant effect on depressive mood (OR = 1.01, 95% CI 0.97–1.06); however, there was a positive significant association with perceived general health (OR = 1.29), perceived happiness (OR = 1.23), and perceived sleep satisfaction (OR = 1.16) and stress (OR = 1.01)
13	Kim et al., 2015 [34], North Korea	Case–control	N = 849 girls	12–18 years old Μean age = 15.0 (SD = 1.5) years old	Food Frequency Questionnaire (FFQ) published by the Korean Health and Nutrition Examination Survey	The Korean version of the Beck Depression Inventory (K-BDI)	Adjustment for energy intake, menstrual regularity	Negative correlation between risk of depression and: - Three servings of green vegetables consumption [*p* = 0.049, OR (95% CI) = 0.61(0.35–1.04)] - One to three servings of fruit per day [*p* = 0.205, OR (95% CI) = 0.56(0.34–0.94), two servings, OR (95% CI) = 0.63(0.37–1.06), three servings]
14	Fisk et al., 2020 [35], United Kingdom	Interventional	N = 64 (45.3% boys)	12–17 years old Mean age = 14.2 (SD = 1.71)	Intervention group: daily supplement of wild blueberry powder in drink for 4 weeks, Control group: placebo fruit drink	Mood and Feelings Questionnaire (MFQ) and Revised Children’s Anxiety and Depression Scale	No confounding factors were examined.	Following the intervention period, there were significantly fewer reported symptoms of depression in participants who received a wild blueberry supplement compared to placebo (*p* = 0.02; 95% CI −6.71, −5.35)

## Data Availability

The original contributions presented in this study are included in the article/Supplementary Materials. Further inquiries can be directed to the corresponding author.

## Supplementary Materials

The following supporting information can be downloaded: https://www.mdpi.com/article/10.3390/children12121617/s1, Table S1. Quality Assessment of the reviewed cross-sectional studies, according to the Newcastle Ottawa Scale. Table S2. Quality Assessment of the reviewed case–control and cohort studies, according to the Newcastle Ottawa Scale. Table S3. Quality Assessment of the reviewed interventional studies, according to the Cochrane ROB2 instrument.

## References

[1] World Health Organization (2013). Mental Health Action Plan 2013–2020. https://www.who.int/publications/i/item/9789241506021.

[2] World Health Organization (2020). Guidelines on Mental Health Promotive and Preventive Interventions for Adolescents: Helping Adolescents Thrive.

[3] UNICEF (2021). Regional Brief: Europe on My Mind. The State of the World ’s Children 2021 Promoting, Protecting and Caring for Children’s Mental Health. https://www.unicef.org/media/108121/file/SOWC-2021-Europe-regional-brief.pdf.

[4] World Health Assembly 65 (2012). Global Burden of Mental Disorders and the Need for a Comprehensive, Coordinated Response from Health and Social Sectors at the Country Level: Report by the Secretariat. https://iris.who.int/handle/10665/78898.

[5] World Health Organization (2023). Depressive Disorder (Depression). https://www.who.int/news-room/fact-sheets/detail/depression.

[6] Jacka F.N., O’Neil A., Opie R., Itsiopoulos C., Cotton S., Mohebbi M., Castle D., Dash S., Mihalopoulos C., Chatterton M.L. (2017). A randomised controlled trial of dietary improvement for adults with major depression (the “SMILES” trial). BMC Med..

[7] Bremner J.D., Moazzami K., Wittbrodt M.T., Nye J.A., Lima B.B., Gillespie C.F., Rapaport M.H., Pearce B.D., Shah A.J., Vaccarino V. (2020). Diet, Stress and Mental Health. Nutrients.

[8] Axelsdóttir B., Biedilæ S., Sagatun Å., Nordheim L.V., Larun L. (2021). Review: Exercise for depression in children and adolescents—A systematic review and meta-analysis. Child. Adolesc. Ment. Health.

[9] Matison A.P., Mather K.A., Flood V.M., Reppermund S. (2021). Associations between nutrition and the incidence of depression in middle-aged and older adults: A systematic review and meta-analysis of prospective observational population-based studies. Ageing Res. Rev..

[10] Głąbska D., Guzek D., Groele B., Gutkowska K. (2020). Fruit and vegetables intake in adolescents and mental health: A systematic review. Rocz. Panstw. Zakl. Hig..

[11] Głąbska D., Guzek D., Groele B., Gutkowska K. (2020). Fruit and Vegetable Intake and Mental Health in Adults: A Systematic Review. Nutrients.

[12] Jacka F.N. (2017). Nutritional Psychiatry: Where to Next?. eBioMedicine.

[13] Adan R.A.H., van der Beek E.M., Buitelaar J.K., Cryan J.F., Hebebrand J., Higgs S., Schellekens H., Dickson S.L. (2019). Nutritional psychiatry: Towards improving mental health by what you eat. Eur. Neuropsychopharmacol..

[14] Lai J.S., Hiles S., Bisquera A., Hure A.J., McEvoy M., Attia J. (2014). A systematic review and meta-analysis of dietary patterns and depression in community-dwelling adults. Am. J. Clin. Nutr..

[15] Saghafian F., Malmir H., Saneei P., Milajerdi A., Larijani B., Esmaillzadeh A. (2018). Fruit and vegetable consumption and risk of depression: Accumulative evidence from an updated systematic review and meta-analysis of epidemiological studies. Br. J. Nutr..

[16] Tuck N.J., Farrow C., Thomas J.M. (2019). Assessing the effects of vegetable consumption on the psychological health of healthy adults: A systematic review of prospective research. Am. J. Clin. Nutr..

[17] Dharmayani P.N.A., Juergens M., Allman-Farinelli M., Mihrshahi S. (2021). Association between Fruit and Vegetable Consumption and Depression Symptoms in Young People and Adults Aged 15–45: A Systematic Review of Cohort Studies. Int. J. Environ. Res. Public Health.

[18] Ventriglio A., Sancassiani F., Contu M.P., Latorre M., Di Salvatore M., Fornaro M., Bhugra D. (2020). Mediterranean Diet and its Benefits on Health and Mental Health: A Literature Review. Clin. Pract. Epidemiol. Ment. Health.

[19] O’Neil A., Quirk S.E., Housden S., Brennan S.L., Williams L.J., Pasco J.A., Berk M., Jacka F.N. (2014). Relationship between diet and mental health in children and adolescents: A systematic review. Am. J. Public Health.

[20] Khanna P., Chattu V.K., Aeri B.T. (2019). Nutritional Aspects of Depression in Adolescents—A Systematic Review. Int. J. Prev. Med..

[21] Page M.J., McKenzie J.E., Bossuyt P.M., Boutron I., Hoffmann T.C., Mulrow C.D., Shamseer L., Tetzlaff J.M., Akl E.A., Brennan S.E. (2021). The PRISMA 2020 statement: An updated guideline for reporting systematic reviews. Syst. Rev..

[22] Winpenny E.M., van Harmelen A.L., White M., van Sluijs E.M., Goodyer I.M. (2018). Diet quality and depressive symptoms in adolescence: No cross-sectional or prospective associations following adjustment for covariates. Public Health Nutr..

[23] Swann O.G., Breslin M., Kilpatrick M., O’Sullivan T.A., Mori T.A., Beilin L.J., Lin A., Oddy W.H. (2021). Dietary fibre intake and its associations with depressive symptoms in a prospective adolescent cohort. Br. J. Nutr..

[24] Yim H.R., Yun H.J., Lee J.H. (2021). An Investigation on Korean Adolescents’ Dietary Consumption: Focused on Sociodemographic Characteristics, Physical Health, and Mental Health. Int. J. Environ. Res. Public Health.

[25] Hong S.A., Peltzer K. (2017). Dietary behaviour, psychological well-being and mental distress among adolescents in Korea. Child. Adolesc. Psychiatry Ment. Health.

[26] Tanaka M., Hashimoto K. (2019). Impact of consuming green and yellow vegetables on the depressive symptoms of junior and senior high school students in Japan. PLoS ONE.

[27] Lv J., Guo X., Meng C., Fei J., Ren H., Zhang Y., Qin Z., Hu Y., Yuan T., Liang L. (2022). The cross-sectional study of depressive symptoms and associated factors among adolescents by backpropagation neural network. Public Health.

[28] Smout S., Gardner L.A., Newton N., Champion K.E. (2023). Dose-response associations between modifiable lifestyle behaviours and anxiety, depression and psychological distress symptoms in early adolescence. Aust. N. Z. J. Public Health.

[29] Kleppang A.L., Haugland S.H., Bakken A., Stea T.H. (2021). Lifestyle habits and depressive symptoms in Norwegian adolescents: A national cross-sectional study. BMC Public Health.

[30] Liang K., Chen S., Chi X. (2022). Care Their Diet and Mind: Association between Eating Habits and Mental Health in Chinese Left-behind Children. Nutrients.

[31] Khayyatzadeh S.S., Omranzadeh A., Miri-Moghaddam M.M., Arekhi S., Naseri A., Ziaee A., Khajavi L., Nejati Salehkhani F., Ferns G.A., Ghayour-Mobarhan M. (2021). Dietary antioxidants and fibre intake and depressive symptoms in Iranian adolescent girls. Public Health Nutr..

[32] Sangouni A.A., Vasmehjani A.A., Ghayour-Mobarhan M., Ferns G.A., Khayyatzadeh S.S. (2022). The association between dietary phytochemical index with depression and quality of life in Iranian adolescent girls. Biopsychosoc. Med..

[33] Park S., Rim S.J., Lee J.H. (2018). Associations between dietary behaviours and perceived physical and mental health status among Korean adolescents. Nutr. Diet..

[34] Kim T.H., Choi Jyoung Lee H.H., Park Y. (2015). Associations between Dietary Pattern and Depression in Korean Adolescent Girls. J. Pediatr. Adolesc. Gynecol..

[35] Fisk J., Khalid S., Reynolds S.A., Williams C.M. (2020). Effect of 4 weeks daily wild blueberry supplementation on symptoms of depression in adolescents. Br. J. Nutr..

[36] Liu M.W., Chen Q.T., Towne SDJr Zhang J., Yu H.J., Tang R., Gasevic D., Wang P.G., He Q.Q. (2020). Fruit and vegetable intake in relation to depressive and anxiety symptoms among adolescents in 25 low- and middle-income countries. J. Affect Disord..

[37] Yang M., Cai C., Yang Z., Wang X., Li G., Li J., Liu J., Zhang Z. (2024). Effect of dietary fibre on cognitive function and mental health in children and adolescents: A systematic review and meta-analysis. Food Funct..

[38] Figueiredo Godoy A.C., Frota F.F., Araújo L.P., Valenti V.E., Pereira E.D.S.B.M., Detregiachi C.R.P., Galhardi C.M., Caracio F.C., Haber R.S.A., Fornari Laurindo L. (2025). Neuroinflammation and Natural Antidepressants: Balancing Fire with Flora. Biomedicines.

[39] Borrego-Ruiz A., Borrego J.J. (2025). Plant-Derived Nutraceuticals in Mental Health and Brain Function: Mechanisms of Action and Therapeutic Potential. Int. J. Mol. Sci..

[40] Fiore M., Terracina S., Ferraguti G. (2025). Brain Neurotrophins and Plant Polyphenols: A Powerful Connection. Molecules.

[41] Agh F., Hasani M., Khazdouz M., Amiri F., Heshmati J., Aryaeian N. (2022). The Effect of Zinc Supplementation on Circulating Levels of Brain-Derived Neurotrophic Factor (BDNF): A Systematic Review and Meta-Analysis of Randomized Controlled Trials. Int. J. Prev. Med..

[42] Punde S., Mandade R.J., Behere S. (2025). Mitochondria-Targeted Nutraceuticals in Depression: Linking Energy Metabolism and Mood. Int. J. Pharm. Sci..

[43] Lu P.M. (2024). Potential Benefits of Specific Nutrients in the Management of Depression and Anxiety Disorders. AMR.

[44] Santhiravel S., Bekhit A.E.-D.A., Mendis E., Jacobs J.L., Dunshea F.R., Rajapakse N., Ponnampalam E.N. (2022). The Impact of Plant Phytochemicals on the Gut Microbiota of Humans for a Balanced Life. Int. J. Mol. Sci..

[45] Dabravolskaj J., Marozoff S., Maximova K., Campbell S., Veugelers P.J. (2022). Relationship Between Fruit and Vegetables Intake and Common Mental Disorders in Youth: A Systematic Review. Public Health Rev..

[46] Jain R., Larsuphrom P., Degremont A., Latunde-Dada G.O., Philippou E. (2022). Association between vegetarian and vegan diets and depression: A systematic review. Nut. Bull..

[47] O’Reilly M., Svirydzenka N., Adams S., Dogra N. (2018). Review of mental health promotion interventions in schools. Soc. Psychiatry Psychiatr. Epidemiol..

[48] Sotiraki M., Malliou A., Tachirai N., Kellari N., Grammatikopoulou M.G., Sergentanis T.N., Vassilakou T. (2022). Burden of Childhood Malnutrition: A Roadmap of Global and European Policies Promoting Healthy Nutrition for Infants and Young Children. Children.

