# Supplementary material for: Association Between Plant Food Consumption and Depressive Symptoms in Adolescents: A Systematic Review
Source: Children (Basel). 2025 Nov 27;12(12):1617. doi: 10.3390/children12121617 (PMC12732234; doi:10.3390/children12121617)
Supplement: Supplementary file 1 [file children-12-01617-s001.zip › children-3963208-supplementary.pdf]

Supplementary Table S1. Quality Assessment of the reviewed cross-sectional studies, according to the Newcastle Ottawa Scale

Study	Selection				Comparability		Outcome		Score
Hong and Pletzer, 2017	*	*	*	*	*	*	*	*	8
Khayyatzadeh et al, 2021	*	*	*	**	*	*	**	*	10
Park et al, 2018	*	*	*	*	*	*	*	*	8
Tanaka et al, 2019	*	*	-	*	*	*	*	*	7
Kleppang et al, 2021	*	*	*	**	*	*	*	*	9
Liang et al, 2022	*	*	*	*	*	*	*	*	8
Lv et al, 2022	*	*	*	**	*	*	**	*	10
Sangouni et al, 2022	-	*	*	**	*	*	**	*	9
Smout et al, 2023	*	*	*	**	*	*	**	*	10

Supplementary Table S2. Quality Assessment of the reviewed case-control and cohort studies, according to the Newcastle Ottawa Scale

Study	Selection				Comparability		Outcome			Score
Case-Control Studies										
Kim et al, 2015	*	*	*	*	*	*	*	*	*	9
Cohort Studies										
Swann et al, 2021	*	*	*	*	*	*	*	*	*	9
Winpenny et al, 2018	*	*	*	*	*	*	*	*	*	9
Yim et al, 2021	*	*	*	*	*	*	*	*	*	9

Supplementary Table S3. Quality Assessment of the reviewed interventional study, according to the Cochrane ROB2 instrument

Study	Randomization	Methods	Missing Data	Outcome	Reporting	Overall Risk of Bias
Fisk et al.,2020	Low	Low	Low	Low	Low	Low
